# High fat diet intake during pre and periadolescence impairs learning of a conditioned place preference in adulthood

**DOI:** 10.1186/1744-9081-7-21

**Published:** 2011-06-26

**Authors:** Gregory J Privitera, Arturo R Zavala, Federico Sanabria, Kristin L Sotak

**Affiliations:** 1 Saint Bonaventure University, Department of Psychology, 3261 West State Street, Saint Bonaventure, NY 14778, USA; 2 California State University, Long Beach, Department of Psychology, 1250 Bellflower Blvd., Long Beach, CA 90840, USA; 3 Arizona State University, Department of Psychology, P.O. Box 871104, Tempe, AZ 85287, USA

## Abstract

**Background:**

Brain regions that mediate learning of a conditioned place preference (CPP) undergo significant development in pre and periadolescence. Consuming a high fat (HF) diet during this developmental period and into adulthood can lead to learning impairments in rodents. The present study tested whether HF diet intake, consumed only in pre and periadolescence, would be sufficient to cause impairments using a CPP procedure.

**Methods:**

Rats were randomly assigned to consume a HF or a low fat (LF) diet during postnatal days (PD) 21-40 and were then placed back on a standard lab chow diet. A 20-day CPP procedure, using HF Cheetos^® ^as the unconditioned stimulus (US), began either the next day (PD 41) or 40 days later (PD 81). A separate group of adult rats were given the HF diet for 20 days beginning on PD 61, and then immediately underwent the 20-day CPP procedure beginning on PD 81.

**Results:**

Pre and periadolescent exposure to a LF diet or adult exposure to a HF diet did not interfere with the development of a HF food-induced CPP, as these groups exhibited robust preferences for the HF Cheetos^® ^food-paired compartment. However, pre and periadolescent exposure to the HF diet impaired the development of a HF food-induced CPP regardless of whether it was assessed immediately or 40 days after the exposure to the HF diet, and despite showing increased consumption of the HF Cheetos^® ^in conditioning.

**Conclusions:**

Intake of a HF diet, consumed only in pre and periadolescence, has long-lasting effects on learning that persist into adulthood.

## Background

Contextual or environmental cues associated with consuming a high fat (HF) food (or an otherwise nutrient rich diet) can influence learning, cognition, food intake, and even override physiological sensations of hunger and fullness [1,2]. In animals, studies using the conditioned place preference (CPP) paradigm, a classic animal model of reward learning, have shown that rats and mice prefer and approach environmental cues that are associated with consumption of a HF food reward. Some HF foods successfully used in CPP studies include open source HF diet pellets [3], fried potatoes [4], corn oil [5], and Cheetos^® ^[6]. In the CPP paradigm, the HF food serves as an unconditioned stimulus (US) that is consumed in a distinct place with a presumably neutral set of environmental cues (the conditioned stimulus, or CS). Over the course of conditioning, the CS acquires secondary motivational properties through a Pavlovian association with the HF food [7,8]. The secondary motivational properties of the CS then elicit an approach response, i.e., when allowed to roam freely, the subject spends most of its time in the presence of the CS instead of a control place.

High consumption of HF foods has unfavorable effects in humans and animals. In humans, a HF diet is associated with obesity [2], and degrading cognitive operation and learning deficits [9], particularly in males [10,11]. Animal studies show impaired learning, spatial memory, memory retention, and hippocampal synaptic plasticity in genetically obese animal models and HF diet-induced obesity models [12-15]. While many studies with rodents begin the HF diets months after weaning [12,16,17], another study began HF diet manipulations immediately following weaning for up to 9 to 12 months thereafter [18]. Whether the observed deficits are due to factors at earlier or later stages of development has not yet been determined.

Of particular interest is the period of adolescence, during which significant brain development occurs [19,20]. In rodents, a conservative definition of adolescence is during postnatal days (PD) 28 to 42 [20,21]; alternative definitions stipulate that adolescence could range from as early as just following weaning to about PD 60 [22,23]. The developmental period from PD 20 to 40 is analogous to preadolescence and adolescence in humans [20]. Adolescence is associated with maturational changes that are evident in mesolimbic and mesocortical brain regions and their terminal regions [24-28]. This maturation is of particular interest here because mesolimbic regions are know to mediate the development of a CPP for a HF Cheetos^® ^food [6,29]. The extent to which a HF diet, consumed only during preadolescence and adolescence, leads to CPP learning deficits has not been investigated.

We hypothesized that HF diet intake during pre and periadolescence would produce long-term learning and memory impairments, possibly due to the significant maturational development that sensitive brain regions undergo during this developmental period. In particular, we expected that learning and memory impairments would be expressed in the failure to acquire CPP in adulthood, even if the US were a highly palatable HF food itself. We did not expect comparable impairments in rats consuming a HF diet at an older age for a comparable period of time or in rats consuming a low fat (LF) diet.

## Methods

### Subjects

Subjects were 30 experimentally naïve, male Sprague-Dawley rats from Charles River Laboratories, Kingston, New York. Male rats were used as they have previously been shown to exhibit a CPP to Cheetos^® ^[6]. Rat pups were housed with their dam and were approximately one-week old upon arrival to the lab. Rats were 21 days old at the start of the study, during which they were separated from the dam and were housed individually in clear plastic cages (43 cm deep × 21 cm wide × 20 cm high). The plastic cages had solid bottoms that were covered with bedding (Sani-Chips, P.J. Murphy Forest Products, Montville, NJ), and the cage tops were stainless steel wire lids. Rats had continuous access to water and lab chow (Harlan Teklad: 2018), except during the 20-day period indicated in the procedures section. Lights were on a 10:14 hour dark:light cycle with lights off at 1130 hours. Both temperature and humidity were controlled in the housing facility, and rats were acclimated to living conditions and handling prior to conducting experimental procedures.

### Apparatus and Diet

The CPP apparatus (26 cm long × 30 cm wide × 32 cm tall, Model H10-11R-TC) had two end chambers of identical size with stainless steel sidewalls and plastic front and back walls. The two end chambers differed on the type of flooring. The floor of one chamber was wire mesh and the floor in the second chamber was aluminum sheet metal gauge 9. No bedding was beneath the floor in either chamber. The two end chambers were connected by a median zone (13 cm long × 23 cm wide × 15.25 cm tall, Model H10-37R-NSF-09W), which also had plastic front and back walls.

During Phase 1 of the experiment, rats consumed a HF diet (60 kcal % fat; Research Diet, Formula D12492) or LF diet (10 kcal % fat; Research Diet, Formula D12450B) for 20 days prior to CPP procedures. In Phase 2 (CPP Training), rats were given access to Cheetos^® ^(56 kcal % fat; Frito-Lay, Inc., Plano, TX) inside the CPP apparatus. Cheetos^® ^were used as a HF US because it is an effective HF food used in CPP studies [6].

### Procedures

Subjects were randomly assigned to one of five groups. During Phase 1, two groups consumed a HF diet and two groups consumed a LF diet during PD 21-40 (Adol HF and Adol LF, respectively). Another group of adult rats consumed the HF diet from PD 61-80 (Adult HF). The adult rats were used as a control group to determine whether any observed CPP deficits were specific to intake during PD 21-40. Groups also differed on whether training (Phase 2) began immediately following Phase 1 (Immediate), or 40 days following Phase 1 (Delayed). Delayed groups were used to determine whether HF diet manipulations induce long-term deficits in CPP performance. The timeline for each group following weaning is given in Table 1, and each phase is summarized here.

**Table 1:** Timeline for the age of rats in each phase by group

Groups	Phase		
	
	1	2	3
	(Diet Manipulation)	(CPP Training)	(CPP Testing)
Adol LF-Immediate (n = 6)	PD 21-40	PD 41-60	PD 61-62
Adol HF-Immediate (n = 6)			
		
Adol LF-Delayed (n = 6)		PD 81-100	PD 101-102
Adol HF-Delayed (n = 6)			

Adult HF - Immediate (n = 6)	PD 61-80	PD 81-100	PD 101-102

All rats were weaned at Postnatal Day (PD) 20. Experimental procedures followed weaning beginning at PD 21 for the Adol LF and Adol HF groups, and beginning at PD 61 for the Adult HF-Immediate group

#### Phase 1 (Diet Manipulation)

Rats in Groups Adol HF-Immediate and Adol HF-Delayed consumed the HF diet ad lib in their home cages for 20 days beginning on PD 21. Groups Adol LF-Immediate and Adol LF-Delayed consumed the LF diet ad lib in their home cages for 20 days beginning on PD 21. Rats in Group Adult HF-Immediate received the HF diet ad lib in their home cages for 20 days beginning on PD 61. Thereafter, all rats consumed a standard lab chow (Harlan Teklad: 2018; 18 kcal % fat) ad lib in their home cages for the duration of the study

#### Phase 2 (CPP Training)

The Adol LF-Immediate and Adol HF-Immediate groups began Phase 2 on PD 41. The Adol LF-Delayed, Adol HF-Delayed, and Adult HF-Immediate groups began Phase 2 on PD 81. Rats were placed in one side of the CPP apparatus for 20 minutes each day for 20 days. Half the rats in each group were given ad lib access to Cheetos^® ^in the wire mesh side on one day and lab chow pellets in the aluminum side on another day; the other half of rats had the reverse pairings. The only HF food consumed during this phase was Cheetos^®^. Five Cheetos^® ^or lab chow pellets were placed on the floor in the center of the chamber. No rat consumed all the Cheetos^® ^or lab chow pellets in any one trial. The difference in the weight of the Cheetos^® ^and pellets from before to after each trial was recorded and converted to kcal for data analysis.

The side a rat was placed in was counterbalanced on an ABBA schedule, where rats were placed in the side associated with the HF food on A-days and the side associated with lab chow pellets on B-days. Because the side that rats received the Cheetos^® ^was chosen at random and was counterbalanced within groups, any individual differences with regard to preferences for one side of the apparatus was assumed to be approximately equal between groups. Meta analysis studies show that this counterbalancing procedure increases effect sizes for a CPP [7].

#### Phase 3 (CPP Testing)

The day after Phase 2 ended, the barrier between each side of the CPP apparatus was removed. No food was placed in either chamber during testing. Each rat was placed in a neutral position in the median zone that separated the two end chambers, and then allowed to roam freely for 10 minutes. Previous studies with 3-chamber CPP systems [30,31] have shown that the amount of time spent in a median zone is small and similar for different groups of rats. Because this was also the case in this study, the meaning of times spent in the median zone was not interpreted.

Data were recorded using a videocamera located above each chamber. The time in each end chamber started when the full body of the rat, excluding the tail, entered the end chamber. Time was stopped when the full body of the rat, excluding the tail, left the end chamber. The amount of time spent in each chamber and number of chamber entrances were recorded. It is important to note that these two dependent measures do not necessarily covary. Each rat was tested for a total of 20 minutes (10 min each day for two consecutive days).

All procedures followed internationally recognized guidelines for ethical conduct in the care and use of animals. The St. Bonaventure University Institutional Animal Care and Use Committee approved all procedures.

### Statistical Analysis

Intake of Cheetos^® ^and lab chow during training trials in Phase 2 were recorded in g, converted to kcal, and analyzed using a mixed-design analysis of variance (ANOVA). Groups and food-side pairings were the between-subjects factors, and days were the within-subjects factor. Post hoc tests were conducted using Tukey's HSD. The dependent variables were amount of Cheetos^® ^and lab chow pellets consumed.

In Phase 3, a CPP was defined as greater time spent in the chamber associated with the HF Cheetos^® ^food compared to time spent in the chamber associated with the lab chow pellets. The preference for the side paired with Cheetos^® ^was measured as the log ratio of time spent in the side paired with Cheetos^® ^to time spent in the side paired with lab chow. The null hypothesis under evaluation in each experimental condition was that the log ratio of the population was zero. Ninety-five and 99% confidence intervals (CIs) were drawn around the log ratios. A CPP was identified when the log ratio was positive and the CI did not envelop zero. Using the same analyses as those in Phase 2, the number of entrances in each chamber was also assessed in Phase 3.

Subject weights were recorded each week in each phase. In each phase subject weights were compared using an analysis of variance (ANOVA) with groups as the between-subjects factor and weeks as the within-subjects factor.

## Results

**Figure 1:**
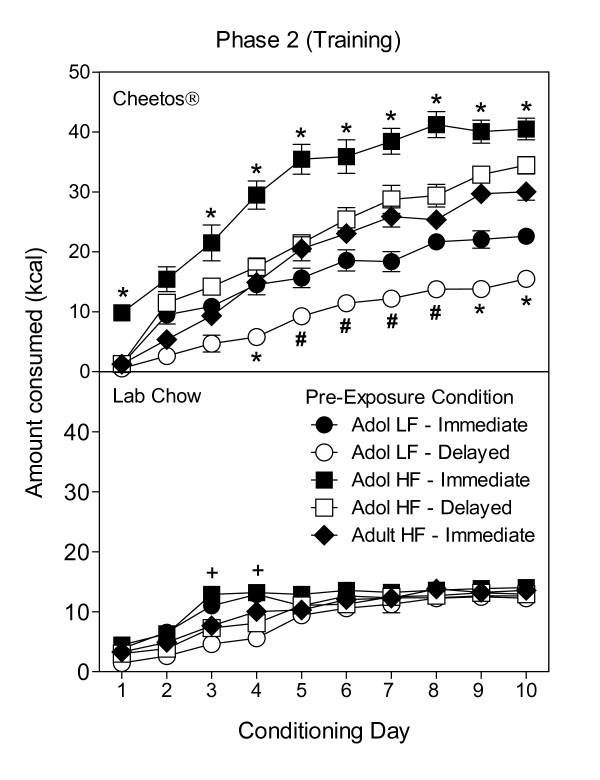
**Mean intake ± SEM of Cheetos^® ^(Top) or Lab chow (Bottom) in kcal consumed during daily 20-min sessions by rats with a history of low fat (LF) or high fat (HF) diet during pre and periadolescence or adulthood**. Access to Cheetos^® ^or lab chow was counterbalanced on an ABBA schedule across 20 days of training (Phase 2). *Significantly different from all other groups (*p *< .05). ^#^Significantly different from all HF groups (*p *< .05). ^+^Significantly different from all LF groups (*p *< .05).

Figure 1 shows the mean consumption of Cheetos^® ^and lab chow across all days for each group in the training phase (Phase 2). The amount of lab chow consumed varied across days, F(9, 225) = 123.42, *p *< .001, and a significant Groups × Days interaction was also evident, F(36, 225) = 2.73, *p *< .001. As illustrated in Figure 1, Tukey's HSD tests showed that the Adol HF-immediate group consumed significantly more lab chow on Days 3 and 4 of conditioning compared to the LF groups (*p *< .05).

Consumption of Cheetos^® ^in Phase 2 significantly varied across days, F(9, 225) = 309.08, *p *< .001, and between groups, F(4, 25) = 51.49, *p *< .001. A significant Groups × Days interaction was also evident, F(36, 225) = 7.35, *p *< .001. As illustrated in Figure 1, Tukey's HSD tests showed that group differences in mean consumption of Cheetos^® ^were significant on virtually every day of conditioning (*p *< .05). Because differences were greatest in the final four days of Phase 2, intake was averaged across the final four Cheetos^® ^days and differences between groups were compared. A one-way between-subjects ANOVA showed a significant main effect of groups, F(4, 25) = 61.84, *p *< .001. Tukey's HSD post hoc tests showed that Group Adol HF-Immediate consumed significantly more Cheetos^® ^than any other group (*p *< .001) over the final four days. Both Adol HF groups consumed significantly more than the Adol LF groups (*p *< .001); and both LF groups consumed significantly less than the Adult HF-Immediate comparison group (*p *< .01).

**Figure 2:**
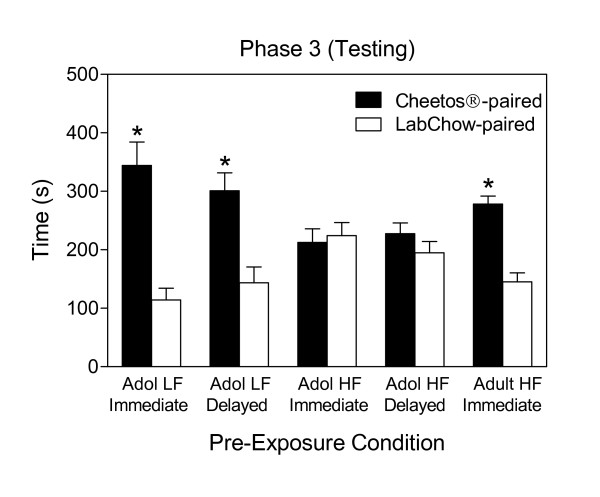
**Mean time ± SEM spent in each chamber during CPP testing (Phase 3) in rats with a history of LF or HF diet during pre and periadolescence or adulthood, and conditioned immediately or after 40 days (delayed) with Cheetos^®^**. An asterisk (*) indicates significance at a 99% CI.

Figure 2 shows the mean time spent in each chamber of the CPP apparatus during testing (Phase 3). Total times spent in the median zone did not differ between groups (*p *> .30). To test for a CPP, time data were converted to log ratios obtained for each rat and 95% and 99% CIs were drawn for each group. Log ratio data did not differ significantly over days, *p *> .17, so the data were pooled across both days of testing. A significant CPP for the side of the apparatus associated with the Cheetos^® ^was evident for Group Adol LF-Immediate (99% CI 0.326, 2.007), Adol LF-Delayed (99% CI 0.078, 1.559), and Adult HF-Immediate (99% CI 0.373, 0.968). A CPP was not observed for Group Adol HF-Immediate (95% CI -0.463, 0.338), and Adol HF-Delay (95% CI -0.181, 0.519). Using 95% CIs, no groups showed a side preference for the left or right side of the cage, indicating that a significant CPP was instead specific to whether a chamber was associated with Cheetos^® ^in Phase 2.

Table 2 shows the means and standard deviations for the number of chamber entrances in each chamber in Phase 3. No significant differences in the number of chamber entrances in Phase 3 were evident across days (*p *> .32) or between groups (*p *> .28).

**Table 2:** The mean and standard deviation for the number of entrances in each side of the CPP apparatus

Group	Cheetos^®^-Paired Side		Lab Chow-Paired Side	
	
	Mean	**St. Dev**.	Mean	**St. Dev**.
Adol LF-Immediate	8.5	3.6	7.4	3.3
Adol HF-Immediate	7.9	2.9	7.4	3.1
Adol LF-Delayed	8.5	3.5	7.0	3.3
Adol HF-Delayed	8.7	2.2	8.3	2.9
Adult HF-Immediate	8.6	3.8	7.9	2.3

**Figure 3:**
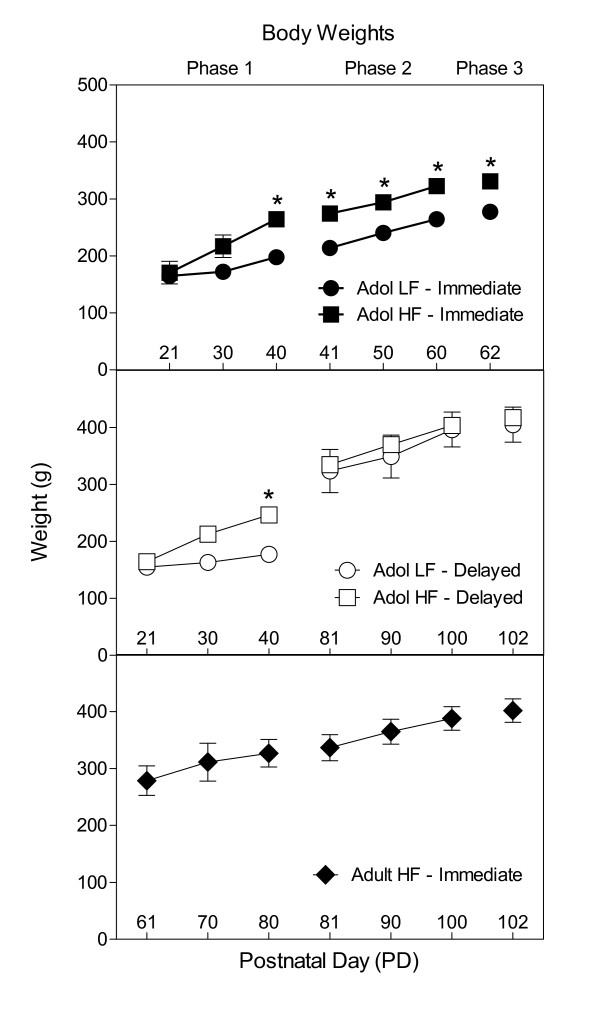
**Mean body weights ± SEM by group during each phase**. Data are separated to make clear that the age of rats was not always the same in each phase. An asterisk (*) indicates significance at *p *< .05.

Figure 3 shows the mean body weights by group in each phase. In Phase 1, rats in the Adol Immediate and Adol Delayed groups were the same age, so weights in these groups were compared. An ANOVA showed a significant main effect of days, F(2, 40) = 185.473, *p *< .001, with weights increasing over days. A Groups × Days interaction was also evident, F(6, 40) = 18.292, *p *< .001. Simple main effect tests showed that rats in the Adol HF groups were significantly heavier than rats in the Adol LF groups (*p *< .001) in Phase 1.

In Phase 2, rats in the two Immediate groups were the same age, so weights in these groups were compared. An ANOVA showed a significant main effect of days, F(2, 20) = 64.937, *p *< .001, and groups, F(2, 20) = 15.573, *p *< .004. Post hoc tests showed that rats in the Adol HF-Immediate group were heavier than the Adol LF-Immediate group each week in Phase 2 (Tukey's HSD, *p *< .001). In Phase 2, rats in the two Delayed groups and the Adult HF-Immediate group were the same age, so weights in these groups were compared. Only a significant main effect of days was evident, F(2, 30) = 116.211, *p *< .001. Overall, weights increased over days (Tukey's HSD, *p *< .001), but did not vary by group in this phase for the Delayed groups and the Adult HF-Immediate group.

In Phase 3, rats in the two Immediate groups were the same age, so weights in these groups were compared. A significant main effect of groups was evident, F(1, 10) = 15.290, *p *< .004, with Group Adol HF-Immediate being significantly heavier than Group Adol LF-Immediate. In Phase 3, rats in the two Delayed groups and Adult HF-Immediate group were the same age, so weights in these groups were compared. The main effect of groups was not significant (*p *> .80), indicating the weights did not vary substantially between the two Delayed groups and the Adult HF-Immediate group in Phase 3.

## Discussion and Conclusions

The present study tested whether intake of a HF diet during the developmental period analogous to preadolescence and adolescence in humans [20] would be sufficient to interfere with a CPP using a HF Cheetos^® ^food as a US. When rats were fed a HF diet during this developmental period (PD 21-40), they consumed the most HF Cheetos^® ^in a subsequent CPP training phase (Phase 2) compared to all other groups. However, these rats did not express a place preference in the CPP testing phase (Phase 3). Moreover, consuming a HF diet for 20 days prior to CPP training in Phase 2 was not sufficient to interfere with a CPP because a CPP was observed in the Adult HF-Immediate group. Thus, only when the HF diet was consumed during PD 21-40 was a CPP no longer evident in Phase 3.

One possible explanation for the results is a negative contrast effect [32] in that the HF diet was so high in fat that, in contrast, the Cheetos^® ^were perceived as being relatively low fat. This possibility is unlikely because the HF diet and the Cheetos^® ^were actually comparable in kcal % fat content: 60 compared to 56 kcal % fat, respectively. In addition, the HF groups consumed significantly more Cheetos^® ^during conditioning in Phase 2 compared to the other three groups. This pattern of responding is not consistent with a negative contrast explanation. An explanation based on motor-related deficits is also unlikely. Groups showed no significant differences in the number of entrances into each side of the CPP apparatus in Phase 3, which suggests comparable locomotion across groups.

Another possible explanation may invoke differences in body weight between groups. Rats in the HF groups were significantly heavier than the LF rats during CPP training and testing. But this was only true for the Immediate groups. After Phase 1, the Delayed groups were placed back on lab chow for 40 days before CPP training and testing began. During this 40-day period, weights normalized. Hence, there were no significant differences in body weight between the Adol HF-Delayed and the Adult HF-Immediate groups at the time of testing, but differences in CPP testing were evident between these groups. These differences in CPP testing make it unlikely that body weight differences explain the results observed in this experiment.

It may be possible that the results reflect a HF diet-induced motivational deficit where, relative to other rats, Adol HF rats "liked" HF food more (i.e., it was more palatable), but "wanted" it less (i.e., sought it less) [33,34]. Rats in the Adol HF groups, however, moved throughout the CPP apparatus in Phase 3 at comparable rates to rats that showed a CPP. It would have been expected that rats that were less motivated to seek HF food would display reduced locomotor activity in the CPP chamber. It is possible, nonetheless, that other factors such as the unconditional locomotor activity elicited by the CPP chamber may have dwarfed the expression of reduced wanting of HF food as locomotor activity. Therefore, selective motivational effects of HF exposure cannot be completely ruled out.

It is also possible that the HF diet consumed during pre and periadolescence may induce a learning deficit. Previous studies have reported learning deficits after intake of a HF diet [12,14,15]. The present study adds to these data by showing that intake of a HF diet for 20 days during the pre and periadolescent period is sufficient to disrupt a CPP using a HF food as a US. Previous studies began the HF diet at least 6 to 8 weeks following weaning and continued the diet for months [12,13,16,17]. This prolonged treatment is similar to the treatment of rats in the Adult HF-Immediate group, except this group consumed a HF diet beginning about 6 weeks post-weaning for 20 days, and not for months. A CPP was evident in this group in Phase 3, indicating that when a HF diet begins after the pre and periadolescence period, 20 days of consuming a HF diet is not sufficient to induce CPP deficits.

A CPP for a place associated with a HF Cheetos^® ^food is known to be mediated by opioids in mesolimbic brain regions [6]. Mesolimbic brain structures and their terminal regions, such as the hippocampus, undergo significant maturation and shifts in midbrain opioid release during pre and periadolescence [33,35-39] and are susceptible to manipulations of fat content in diet [9,40]. These brain structures may mediate a CPP, however further research is needed to test this hypothesis because many other biochemical and neurobiological factors related to changes in body weight and appetite regulation may also mediate a CPP using a HF US. These factors include possible HF-diet induced changes in leptin [41,42], ghrelin [3,43], neuropeptide Y [44,45], agouti-related protein (AGRP) [46], and proopiomelanocortin (POMC) [47]--none of which were measured in this study.

The behavioral pattern observed in the present study is similar to that for human infants with salt. Studies show that 16-week-old human infants of mothers who reported frequent or severe vomiting expressed stronger preferences for salty solutions compared to 16-week-old infants from mothers reporting little to no vomiting [48]. This difference in salt preferences appears to persist at least until adolescence [49]. Hence, when the amnion is deplete of salt, mechanisms that control liking for salt are enhanced, thereby ensuring that this presumably scarce nutrient is consumed when it is found. Similarly, in this study LF rats showed significant learning for a place associated with a HF food, whereas HF-Adol rats did not. During development, fat was a plentiful nutrient for the HF-Adol rats and it was a scare nutrient for the LF rats. Hence, it would be advantageous for the LF rats to show a place preference for the "scarce" fat nutrient, but not for the HF-Adol rats because fat was a presumably "abundant" nutrient. The fact that CPP learning was not expressed even in the HF-Delayed group that had 40 days of a lab chow diet, suggests that whatever mechanisms of learning were affected, the deficits may be long-lasting. This interpretation of the results, however, suggests that HF food-induced CPP learning deficits may be specific to HF USs. Whether these deficits generalize to other USs is yet to be established.

Aside from only considering one type of US during CPP--Cheetos--the present study is also limited to one sex (male), a limited duration of exposure (20 days during adolescence), and to a particular HF diet (60 kcal % fat). Although it is yet unknown whether the present results generalize to female rats, the differential impact of a HF diet on cognition and learning across sexes [10,11] suggests that such generalization should not be taken for granted. In addition, it is possible that the critical developmental period for HF diet effects is a shorter interval within adolescence (e.g., early adolescence), but further research is necessary to identify such a period. At present, this study demonstrates that 20 days of consuming a HF diet is sufficient to interfere with a CPP, but only when the HF diet is implemented immediately following weaning. It was established, however, that the observed deficits are long-term, lasting well into adulthood, as evidenced by the failure of Group Adol HF-Delayed to show evidence of a CPP. These results indicate that pre and periadolescence are critical periods for producing long-term CPP deficits resulting from HF diet intake.

## Competing interests

The authors declare that they have no competing interests.

## Authors' contributions

GJP designed and performed the experiment, analyzed the data, and wrote the manuscript. ARZ and FS co-designed the experiment, analyzed the data, and provided important revisions in the writing of the manuscript. KLS assisted in performing the experiment, measuring data, and reviewing the manuscript. All authors read and approved the final manuscript.

## References

[B1] Davidson TL Benoit SC O'Donohue WT Learning and eating Learning and Behavior Therapy 1998 Reno, NV: Prentice Hall 498 517

[B2] Privitera GJ The psychological dieter: It's not all about the calories 2008 Lanham, MD: University Press of America21536333

[B3] Perello M Sakata I Birnbaum S Chuang JC Osborne-Lawrence S Rovinsky SA Woloszyn J Yanagisawa M Lutter M Zigman JM Ghrelin increases the rewarding value of high-fat diet in an orexin-dependent manner Biological Psychiatry 2010 67 880 886 10.1016/j.biopsych.2009.10.030 20034618 PMC2854245

[B4] Imaizumi M Takeda M Suzuki A Sawano S Fushiki T Preference for high-fat food in mice: Fried potatoes compared with boiled potatoes Appetite 2001 36 237 238 10.1006/appe.2001.0399 11358348

[B5] Matsumura S Yoneda T Aki S Eguchi A Manabe Y Tsuzuki S Inoue K Fushiki T Intragastric infusion of glucose enhances the rewarding effect of sorbitol fatty acid ester ingestion as measured by conditioned place preference in mice Physiology & Behavior 2010 99 509 514 10.1016/j.physbeh.2009.12.018 20045421

[B6] Jarosz PA Sekhon P Coscina DV Effect of opioid antagonism on conditioned place preferences to snack foods Pharmacology, Biochemistry, and Behavior 2006 83 257 264 10.1016/j.pbb.2006.02.004 16540156

[B7] Bardo MT Rowlett JK Harris MJ Conditioned place preference using opiate and stimulant drugs: A meta-analysis Neuroscience and Biobehavioral Reviews 1995 19 39 51 10.1016/0149-7634(94)00021-R 7770196

[B8] Tzschentke TM Measuring reward with the conditioned place preference paradigm: A comprehensive review of drug effects, recent progress and new issues Progress in Neurobiology 1998 56 613 672 10.1016/S0301-0082(98)00060-4 9871940

[B9] Beydoun MA Beydoun HA Wang Y Obesity and central obesity as risk factors for incident dementia and its subtypes: A systematic review and meta-analysis Obesity Reviews 2008 9 204 218 10.1111/j.1467-789X.2008.00473.x 18331422 PMC4887143

[B10] Elias MF Elias PK Sullivan LM Wolf PA D'Agostino RB Lower cognitive function in the presence of obesity and hypertension: The Framington heart study International Journal of Obesity 2003 27 260 268 10.1038/sj.ijo.802225 12587008

[B11] Gustafson D Rothenberg E Blennow K Steen B Skoog I An 18-year follow-up of overweight and risk of Alzheimer disease Archives of Internal Medicine 2003 163 1524 1528 10.1001/archinte.163.13.1524 12860573

[B12] Kanoski SE Davidson TL Different patterns of memory impairments accompany short- and longer-term maintenance on the high-energy diet Journal of Experimental Psychology: Animal Behavior Processes 2010 36 313 319 20384410 10.1037/a0017228

[B13] Martin B Pearson M Kebejian L Golden E Keselman A Bender M Carlson O Egan J Ladenheim B Cadet JL Becker KG Wood W Duffy K Vinayakumar P Maudsley S Mattson MP Sex-dependent metabolic, neuroendocrine, and cognitive responses to dietary energy restrictions and excess Endocrinology 2007 148 4318 4333 10.1210/en.2007-0161 17569758 PMC2622430

[B14] Gerges NZ Aleisa M Alkadhi KA Impaired long-term potentiation in obese Zucker rats: Possible involvement of presynaptic mechanism Neuroscience 2003 120 535 539 10.1016/S0306-4522(03)00297-5 12890522

[B15] Li XL Aou S Oomura Y Hori N Fukunaga K Hori T Impairment of long-term potentiation and spatial memory in leptin receptor-deficient rodents Neuroscience 2002 113 607 615 10.1016/S0306-4522(02)00162-8 12150780

[B16] Baladi MG France CP High fat diet and food restriction differentially modify the behavioral effects of quinpirole and raclopride in rats European Journal of Pharmacology 2009 610 55 60 10.1016/j.ejphar.2009.03.048 19327348 PMC2697565

[B17] Lassiter TL Ryde IT Levin ED Seidler FJ Slotnik TA Neonatal exposure to parathion alters lipid metabolism in adulthood: Interactions with dietary fat intake and implications for neurodevelopmental deficits Brain Research Bulletin 2010 81 85 91 10.1016/j.brainresbull.2009.07.002 19615431 PMC2795103

[B18] Hwang LL Wang CH Li TL Chang SD Lin LC Chen CP Chen CT Liang KC Ho IK Yang WS Chiou LC Sex differences in high-fat diet-induced obesity, metabolic alterations and learning, and synaptic plasticity deficits in mice Obesity 2010 18 463 469 10.1038/oby.2009.273 19730425

[B19] Jameson JL De Groot LJ Endocrinology, 2-volume set: Adult and pediatric, expert consult premium edition 2010 6 New York: Saunders

[B20] Spear LP The adolescent brain and age-related behavioral manifestations Neuroscience and Biobehavioral Reviews 2000 24 417 463 10.1016/S0149-7634(00)00014-2 10817843

[B21] Spear LP Brake SC Periadolescence: Age-dependent behavior and psychopharmacological responsivity in rats Developmental Psychobiology 1983 16 83 109 10.1002/dev.420160203 6339302

[B22] Odell WD Grumbach MM, Sizonenko PC, Aubert ML Sexual maturation in the rat Control of the Onset of Puberty 1990 Baltimore, MD: Williams and Wilkins 183 210

[B23] Ojeda SR Urbanski HF Knobil E, Neill JD Puberty in the rat The Physiology of Reproduction 1994 2 New York, NY: Raven Press 363 409

[B24] Akbari HM Kramer HK Whitaker-Azmitia PM Spear LP Azmitia EC Prenatal cocaine exposure disrupts the development of the serotonergic system Brain Research 1992 572 57 63 10.1016/0006-8993(92)90450-N 1535274

[B25] Andersen SL Dumont NL Teicher MH Developmental differences in dopamine synthesis inhibition by (±)-7-OH-DPAT Naunyn-Schmiedeberg's Archives of Pharmacology 1997 356 173 181 10.1007/PL00005038 9272722

[B26] Leslie CA Robertson MW Cutler AJ Bennett JP Jr Postnatal development of D1 dopamine receptors in the medial prefrontal cortex, striatum, and nucleus accumbens of normal and neonatal 6-hydroxydopamine treated rats: A quantitative autoradiographic analysis Developmental Brain Research 1991 62 109 114 10.1016/0165-3806(91)90195-O 1836980

[B27] Tarazi FI Tomasini EC Baldessarini RJ Postnatal development of dopamine D4-like receptors in rat forebrain regions: Comparison with D2-like receptors Developmental Brain Research 1998 110 227 233 10.1016/S0165-3806(98)00111-4 9748595

[B28] Tarazi FI Tomasini EC Baldessarini RJ Postnatal development of dopamine D1-like receptors in rat cortical and striatolimbic brain regions: An autoradiographic study Developmental Neuroscience 1999 21 43 49 10.1159/000017365 10077701

[B29] Kiozumi M Cagniard B Murphy NP Endogenous nociceptin modulates diet preference independent of motivation and reward Physiology & Behavior 2009 97 1 13 10.1016/j.physbeh.2008.12.008 19138695

[B30] Bechara A van der Kooy D A single brain stem substrate mediates the motivational effects of both opiates and food in nondeprived rats but not in deprived rats Behavioral Neuroscience 1992 106 351 363 1317187 10.1037//0735-7044.106.2.351

[B31] Bechara A Harrington F Nader K van der Kooy D Neurobiology of motivation: Double dissociation of two motivational mechanisms mediating opiate reward in drug-naïve versus drug dependent animals Behavioral Neuroscience 1992 106 798 807 1359898 10.1037//0735-7044.106.5.798

[B32] Flaherty CF Checke S Anticipation of incentive gain Animal Learning and Behavior 1992 10 177 182

[B33] Kelley AE Berridge KC The neuroscience of natural rewards: Relevance to addictive drugs The Journal of Neuroscience 2002 22 3306 3311 11978804 10.1523/JNEUROSCI.22-09-03306.2002 PMC6758373

[B34] Tindell AJ Smith KS Berridge KC Aldridge JW Dynamic computation of incentive salience: "Wanting" what was never "liked." The Journal of Neuroscience 2009 29 12220 12228 10.1523/JNEUROSCI.2499-09.2009 19793980 PMC2792765

[B35] Bodnar RJ Endogenous opioids and feeding behavior: A 30-year historical perspective Peptides 2004 25 697 725 10.1016/j.peptides.2004.01.006 15165728

[B36] Yeomans MR Gray RW Opioid peptides and the control of human ingestive behaviour Neuroscience & Biobehavioral Reviews 2002 26 713 728 10.1016/S0149-7634(02)00041-6 12479844

[B37] Floresco SB Blaha CD Yang CR Phillips AG Modulation of hippocampal and amygdalar-evoked activity of nucleus accumbens neurons by dopamine: Cellular mechanisms of input selection The Journal of Neuroscience 2001 21 2851 2860 11306637 10.1523/JNEUROSCI.21-08-02851.2001 PMC6762526

[B38] Legault M Wise RA Novelty-evoked elevations of nucleus accumbens dopamine: Dependence on impulse flow from the ventral subiculum and glutamatergic neurotransmission in the ventral tegmental area The European Journal of Neuroscience 2001 13 819 828 10.1046/j.0953-816x.2000.01448.x 11207817

[B39] Mulder AB Hodenpijl MG Lopes da Silva FH Electrophysiology of the hippocampal and amygdaloid projections to the nucleus accumbens of the rat: Convergence, segregation, and interaction of inputs The Journal of Neuroscience 1998 18 5095 5102 9634575 10.1523/JNEUROSCI.18-13-05095.1998 PMC6792568

[B40] Dumas TC Foster TC Late developmental changes in the ability of adenosine A1 receptors to regulate synaptic transmission in the hippocampus Developmental Brain Research 1998 105 137 139 10.1016/S0165-3806(97)00152-1 9473633

[B41] Zhang C Su Z Zhao B Qu Q Tan Y Cai L Li X Tat-modified leptin is more accessible to hypothalamus through brain-blood barrier with a significant inhibition of body-weight gain in high-fat-diet fed mice Experimental and Clinical Endocrinology & Diabetes 2010 118 31 37 10.1055/s-0029-1202273 19472101

[B42] Glavas MM Kirigiti MA Xiao XQ Enriori PJ Fisher SK Evans AE Grayson BE Cowley MA Smith MS Grove KL Early overnutrition results in early-onset arcuate leptin resistance and increased sensitivity to high-fat diet Endocrinology 2010 151 1598 1610 10.1210/en.2009-1295 20194730 PMC2850236

[B43] Wang ZQ Zuberi AR Zhang XH Macgowan J Qin-J Ye X Son L Wu Q Lian K Cefalu WT Effects of dietary fibers on weight gain, carbohydrate metabolism, and gastric ghrelin gene expression in mice fed a high-fat diet Metabolism: Clinical and Experimental 2007 56 1635 1642 10.1016/j.metabol.2007.07.004 PMC2730183 17998014

[B44] Morris MJ Chen H Watts R Shulkes A Cameron-Smith D Brain neuropeptide Y and CCK and peripheral adipokine receptors: temporal response in obesity induced by palatable diet International Journal of Obesity 2008 32 249 258 10.1038/sj.ijo.0803716 17768423

[B45] Hollopeter G Erickson JC Palmiter RD Role of neuropeptide Y in diet-, chemical-, and genetic-induced obesity of mice International Journal of Obesity and Related Metabolic Disorders 1998 22 506 512 10.1038/sj.ijo.0800615 9665670

[B46] Staszkiewicz J Horswell R Argyropoulos G Chronic consumption of a low-fat diet leads to increased hypothalamic agouti-related protein and reduced leptin Nutrition 2007 23 665 671 10.1016/j.nut.2007.06.001 17643264 PMC2030621

[B47] Horvath TL Sarman B Garcia-Caceres C Enriori PJ Sotonyi P Shanabrough M Borok E Argente J Chowen JA Perez-Tilve D Pfluger PT Bronneke HS Levin BE Diano S Cowley MA Tschop MH Synaptic input organization of the melanocortin system predicts diet-induced hypothalamic reactive gliosis and obesity Proceedings of the National Academy of Sciences of the United States of America 2010 107 14875 80 10.1073/pnas.1004282107 20679202 PMC2930476

[B48] Crystal SR Bernstein IL Infant salt preference and mother's morning sickness Appetite 1998 30 297 307 10.1006/appe.1997.0144 9632460

[B49] Leshem M Salt preference in adolescence is predicted by common prenatal and infantile mineralofluid loss Physiology & Behavior 1998 63 699 704 10.1016/S0031-9384(97)00525-8 9523918

